# Short-term safety and feasibility of a practical approach to combined atrial and ventricular physiological pacing: An initial single-center experience

**DOI:** 10.1016/j.hroo.2024.04.002

**Published:** 2024-05-16

**Authors:** Keita Watanabe, Moritz Nies, William Whang, Chengyue Jin, Abhishek Mann, Daniel Musikantow, Joshua Lampert, Marc Miller, Mohit Turagam, Samuel Hannon, Marie-Noelle Langan, Srinivas Dukkipati, Vivek Y. Reddy, Jacob Koruth

**Affiliations:** Helmsley Electrophysiology Center, Department of Cardiology, Icahn School of Medicine at Mount Sinai, New York, New York

**Keywords:** Atrialmyocardium, Bachmann bundle, Interatrial septum, Interventricular septum, Left bundle, Pacing, Right atrial appendage

## Abstract

**Background:**

Traditional right atrial appendage (RAA) pacing accentuates conduction disturbances as opposed to Bachmann bundle pacing (BBP).

**Objective:**

The purpose of this study was to evaluate the feasibility, efficacy, and safety of routine anatomically guided high right atrial septal (HRAS) pacing with activation of Bachmann bundle combined with routine left bundle branch area pacing (LBBAP).

**Methods:**

This retrospective single-center study included 96 consecutive patients who underwent 1 of 2 strategies: physiological pacing (PP) (n = 32) with HRAS and LBBAP leads and conventional pacing (CP) (n = 64) with traditional RAA and right ventricular apical leads. Baseline characteristics, sensing, pacing thresholds, and impedances were recorded at implantation and follow-up.

**Results:**

The PP and CP cohorts were of similar age (74.2 ± 13.8 years vs 73.9 ± 9.9 years) and sex (28.1% vs 40.6% female). There were no differences in procedural time (95.0 ± 31.4 minutes vs 86.5 ± 33.3 minutes; *P* = .19) or fluoroscopy time (12.1 ± 4.5 minutes vs 12.3 ± 13.5 minutes; *P* = .89) between cohorts. After excluding patients who received >2 leads, these parameters became significantly shorter in the CP cohort. The PP cohort exhibited higher atrial pacing thresholds (1.5 ± 1.1 mV vs 0.8 ± 0.3 mV; *P* <.001) and lower p waves (1.8 ± 0.8 mV vs 3.8 ± 2.3 mV; *P* <.001) at implantation and at follow-up. In the PP cohort, 72% of implants met criteria for BBP; of the ventricular leads, 94% demonstrated evidence of LBBAP. One lead-related complication occurred in each cohort.

**Conclusion:**

Routine placement of leads in the HRAS is a feasible and safe alternative to standard RAA pacing, allowing for BBP in 72% of patients. HRAS pacing can be combined with LBBAP as a routine strategy.

**Key Findings**

▪ Bachmann pacing was achieved in 72% of patients with atrial lead placement in the high right atrial septum.
▪ Routine placement of pacing leads in the high right atrial septum is a feasible and safe alternative to standard right atrial appendage pacing.
▪ A combination of high right atrial septal pacing and left bundle branch area pacing can be used as a routine strategy.

## Introduction

The right ventricular (RV) apex and right atrial appendage (RAA) have traditionally been considered optimal fixation sites for delivering tined leads because of their trabeculated and narrow anatomy. These sites, although essential for stable fixation of tined lead tips, continue to be favored as implant locations for active fixation leads as well. In recent years, left bundle branch area pacing (LBBAP) has led to a shift away from RV apical pacing given the benefits of physiological ventricular activation. This has reduced left ventricular dyssynchrony and has provided important advantages such as elimination of lead tip–related RV perforation and tamponade.^1^^,^^2^

Similar to RV apical pacing, RAA pacing accentuates interatrial conduction delay, which in turn can predispose to atrial arrhythmias over time. Furthermore, the thin-walled RAA risks lead perforation, especially in the elderly.^3^ The high right atrial septum (HRAS) is an alternative implant site that has the potential to not only improve biatrial activation but also eliminate perforation and consequent pericarditis and/or tamponade. Specific pacing locations in the high atrial septum have been described to allow for capture of the Bachmann bundle as evidenced by specific changes in P-wave characteristics. Bachmann pacing compared to RAA pacing has been shown in recent reports to reduce atrial fibrillation.^4^^,^^5^ However, its uptake in clinical practice has not seen the same enthusiasm as has been witnessed for LBBAP.

In this report, we present our initial experience with routine use of HRAS pacing for all patients requiring atrial leads at our institution, focusing on feasibility, efficacy, and safety. For ventricular pacing or sensing, we also performed LBBAP routinely for all-comers so as to provide the most physiological dual-chamber pacing strategy. Here we report procedural parameters and early follow-up outcomes for this combined strategy.

## Methods

### Patient selection

This retrospective study consists of 2 cohorts from a single center. Thirty-two consecutive patients between December 2022 and July 2023 who met standard pacing indications and planned for physiological pacing of the right atrium and ventricle were included as the study cohort (physiological pacing [PP] cohort) after having provided consent to the implant procedure. The SelectSecure lead (Model 3830, Medtronic Inc., Minneapolis, MN) was used for both atrial and ventricular pacing in all patients and was delivered through a deflectable sheath or fixed curve sheaths (C315 HIS, C304His, C315-J, and C315-S5, Medtronic). The atrial lead was implanted in the high right atrial septum targeting the Bachmann bundle and for the ventricle was implanted in the left bundle branch (LBB) area. A control cohort consisted of 64 consecutive patients who underwent atrial lead implantation in the RAA and a lead in the RV apex during the same time period (conventional pacing [CP] cohort). In this cohort, any stylet-driven active or passive fixation leads from any manufacturer were allowed as long as a lead was implanted in the RAA and RV apex (including defibrillator leads). In order to minimize selection bias and to allow the cohorts to represent all-comers presenting to the electrophysiology laboratory, we did not exclude cardiac resynchronization devices in either cohort and allowed dual-chamber defibrillators in the control group. This study was approved by the Institution Review Board at the Mount Sinai Hospital. The research reported in this paper adhered to the Helsinki Declaration as revised in 2013. Ethical committee approval was obtained.

### PP cohort details

#### HRAS pacing

**Figure 1:**
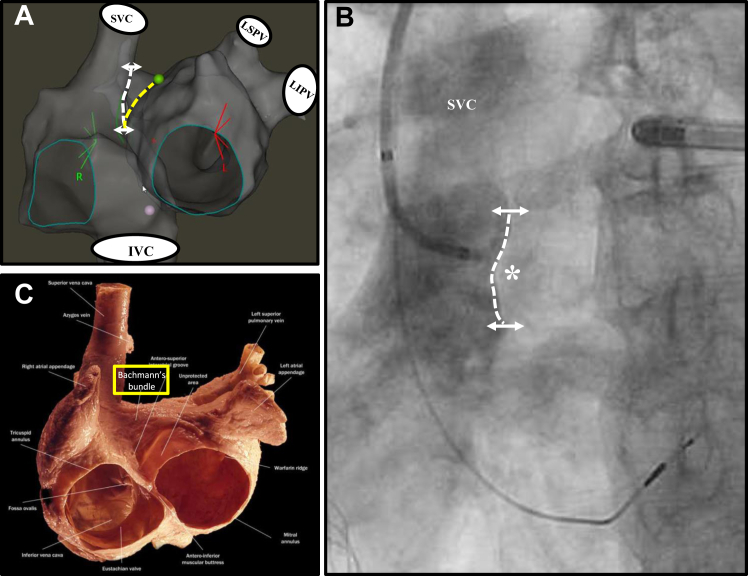
High right atrial septal anatomy. **A:** Electroanatomic maps of right atrium and left atrium (LA) seen in the left anterior oblique (LAO) projection. *White dotted line* outlines the high right atrial septum. *Yellow dotted line* represents the endocardial border of the septal LA. **B:***White dotted line* corresponds to the septal aspect of the contrast-filled superior vena cava (SVC)–right atrial junction seen in LAO projection. The typical target site for lead fixation is the mid-portion (∗) or just below this along the *white dotted line*. **C:** The epicardial structure overlying this region is where the Bachmann bundle is located and is highlighted by the *yellow box*. Atlas of Cardiac Anatomy, Digital Edition © 2022 Shumpei Mori, Kalyanam Shivkumar. IVC = inferior vena cava; LIPV = left inferior pulmonary vein; LSPV = left superior pulmonary vein.

**Figure 2:**
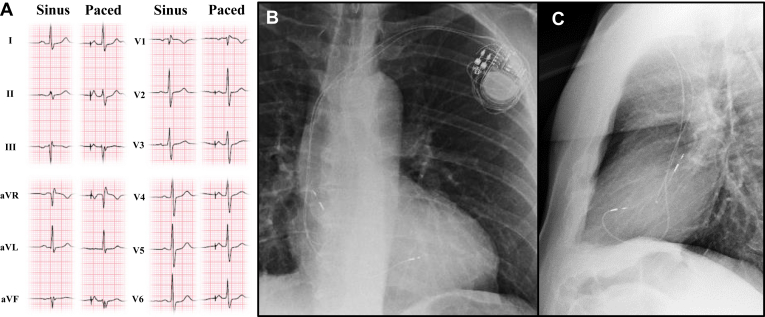
P-wave characteristics of Bachmann bundle pacing. **A:** Sinus rhythm and high right atrial paced rhythm from a patient in the high right atrial septal pacing cohort who met all criteria for Bachmann bundle pacing. Note the tall and narrow P waves best appreciated in the inferior leads. **B, C:** Chest radiographs in posteroanterior **(B)** and lateral **(C)** views showing atrial lead position in the same patient and the ventricular lead in the typical position for left bundle branch area pacing.

Specific steps related to this procedure involved contrast injection through the delivery sheath positioned in the superior vena cava in the left anterior oblique view so as to identify the high septal concavity (Figure 1).^6^ The lead was then advanced through the sheath, and atrial sensing was assessed in unipolar mode in the mid to lower portion of this high septal concavity. The right anterior oblique view was used to ensure that the lead tip was not directed anteriorly. Sensing values >1 mV and thresholds <1 mV at 0.5-ms pulse width were considered acceptable. Pacing thresholds >1 mV at 0.5-ms pulse width were deemed acceptable only if accompanied by a prominent current of injury at the time of implant. During the procedure, repositioning of the lead was performed to allow for optimal P-wave morphology (Bachmann bundle pacing; Figure 2) and/or acceptable pacing and sensing parameters. A 12-lead electrocardiographic (ECG) recording system was used for all procedures.

Postoperative posteroanterior and lateral chest radiographs obtained the next morning were reviewed for all patients to confirm stability of lead placement. P-wave morphology was assessed by continuous 12-lead ECGs recorded at double gain during threshold testing on the first postoperative day and measured using ImageJ software (National Institute of Health) electronically. For patients who received LBBAP in the ventricle, threshold testing of this lead was also performed on the 12-lead ECG to assess for QRS morphology changes related to LBB capture. Determination of Bachmann bundle pacing was made retrospectively using published criteria.^5^^,^^7^ These include (1) P-wave axis similar to that of normal sinus rhythm, with upright P waves in leads I, II, III, and aVF and biphasic or negative P waves in lead V_1_; (2) peaked and symmetric P waves in inferior limb leads with paced P-wave amplitude typically greater than sinus P-wave amplitude; (3) P-wave duration narrower than sinus P-wave duration, typically by >10 ms if baseline interatrial conduction delay was present (Figure 2).

In the PP group, 2 electrophysiologists (JK, KW) retrospectively determined whether all 3 P-wave criteria were met and assigned the subjects to either the Bachmann pacing group or non–Bachmann pacing group.

#### LBBAP (PP cohort)

Briefly, LBBAP was performed (SelectSecure Model 3830, 69 cm, Medtronic) and dedicated delivery sheaths (C315HIS, C304, Medtronic). A basal to mid-interventricular septal site was selected ∼1.5–2 cm apical from the distal His location with appropriate paced morphology as has been described.^1^, ^2^ Lead deployment was performed under fluoroscopy, continuous ECG, and unipolar and bipolar electrogram sensing and pacing guidance. We aimed to obtain a paced QRS complex with an r′ deflection in lead V_1_ and features of LBB capture. Attempts were made to obtain LBB capture, but left ventricular septal pacing was considered acceptable.

Detailed pacing and sensing parameters were obtained during follow-up, and all assessments, including the most recent, were included. Baseline characteristics including transthoracic echocardiographic data and fluoroscopy and procedural durations were collected for all patients. All patient records were reviewed carefully to adjudicate procedure-related complications and device-related interventions in follow-up.

### Statistical analysis

Differences in baseline clinical characteristics and echocardiographic parameters were compared using the Student *t* test for parametric data and Mann-Whitney *U* test for nonparametric data. Categorical variables were compared using the χ^2^ test or Fisher exact test. Statistical analyses were performed using SPSS 24.0 software (SPSS, Inc., Chicago, IL). Baseline demographic, clinical, ECG, and procedural data were collected by chart review.

## Results

A total of 96 patients were included in the study (32 in the PP cohort and 64 in the CP cohort). Mean follow-up in this evaluation was 71.6 ± 69.6 days after implant for the PP cohort and 74.9 ± 54.8 days for CP cohort (*P* = .836).

### Baseline characteristics

Mean age of the PP cohort was 74.2 ± 13.8 years and 71.9% were male, which was not statistically different from the CP cohort, whose mean age was 73.9 ± 9.9 years and 59.4% were male (Table 1). Both cohorts exhibited no significant differences in baseline characteristics, including the presence of hypertension, heart failure, coronary disease, and use of medications. There were no significant differences in echocardiographic parameters such as left ventricular ejection fraction, end-diastolic diameter, and right and left atrial sizes between both cohorts. Of the entire cohort, 71.9% underwent dual-chamber pacemaker implantation; 60.4% for atrioventricular block–related bradycardia and 14.6% for sick sinus syndrome. The remaining cohort included patients who received dual-chamber (25.0%) or biventricular cardioverter-defibrillators (16.7%) for primary/secondary prevention (19.8% and 5.2%) and 3.1% who received biventricular pacemakers (Table 1). There were no significant differences between the 2 cohorts in terms of the indication for devices implanted.

**Table 1:** Patients characteristics

	All	PP	CP	*P* value	PP excluding CRT	CP excluding CRT	*P* value
	(n = 96)	(n = 32)	(n = 64)		(n = 28)	(n = 48)	
Age (y)	74.0 ± 11.4	74.2 ± 13.8	73.9 ± 9.9	.902	74.8 ± 14.7	75.1 ± 9.2	.908
Female	35 (36.5)	9 (28.1)	26 (40.6)	.267	9 (32.1)	20 (41.7)	.469
HTN	74 (77.1)	27 (84.4)	47 (73.4)	.206	24 (85.7)	37 (77.1)	.551
DM	28 (29.2)	14 (43.8)	14 (21.9)	.0388	11 (39.3)	10 (20.8)	.112
CAD	47 (49.0)	16 (50.0)	31 (48.4)	.887	13 (46.4)	25 (52.1)	.812
HF	35 (36.5)	9 (28.1)	26 (40.6)	.223	5 (17.9)	12 (25.0)	.575
AF	27 (28.1)	8 (25.0)	19 (29.7)	.63	6 (21.4)	15 (31.3)	.432
Echocardiography							
EF (%)	53.0 ± 14.8	55.8 ± 14.5	51.6 ± 14.7	.214	59.8 ± 11.1	58.5 ± 8.5	.616
LVEDD (mm)	47.9 ± 8.2	48.3 ± 9.1	47.6 ± 7.7	.752	46.6 ± 8.6	45.0 ± 5.6	.433
LA volume (mL)	70.3 ± 26.7	74.0 ± 25.9	68.4 ± 26.9	.366	74.8 ± 26.7	69.6 ± 28.6	.459
RA volume (mL)	49.3 ± 22.5	55.8 ± 28.1	45.9 ± 18.1	.135	55.9 ± 29.2	45.8 ± 19.5	.178
Indication							
AV block	58 (60.4)	21 (65.6)	37 (57.8)	.512	21 (75.0)	34 (70.8)	.794
SSS	14 (14.6)	6 (18.8)	8 (12.5)	.363	6 (21.4)	8 (16.7)	.760
Primary prevention	19 (19.8)	5 (15.6)	14 (21.9)	.591	1 (3.6)	2 (4.2)	1
Secondary prevention	5 (5.2)	0	5 (7.8)	.166	0	4 (8.3)	.290
Type of device							
Dual PM	69 (71.9)	28 (87.5)∗	41 (64.1)†	.0172	28 (100)	41 (85.4)	.0425
Dual ICD	7 (7.3)	0 (0)	7 (10.9)	.0921	0	7 (14.6)	.0425
CRT-D	17 (17.7)	4 (12.5)‡	13 (20.3)§	.408	0	0	
CRT-P	3 (3.1)	0 (0)	3 (4.7)§	.548	0	0	

Values are given as mean ± SD or n (%) unless otherwise indicated.

AF = atrial fibrillation; AV = atrioventricular; CAD = coronary artery disease; CP = conventional pacing; CRT = cardiac resynchronization therapy; CRT-D = cardiac resynchronization therapy–defibrillator; CRT-P = cardiac resynchronization therapy–pacemaker; DM = diabetes mellitus; EF = ejection fraction; HF = heart failure; HTN = hypertension; ICD = implantable cardioverter-defibrillator; LA = left atrium; LBBA = left bundle branch area; LVEDD = left ventricular end-diastolic dimension; PM = pacemaker; PP = physiological pacing; RA = right atrium; SSS = sick sinus syndrome.

∗ Dual-chamber pacemakers in the PP group had atrial and ventricular leads in the high right atrial septal and LBBA positions.

‡ CRT-D devices in the PP group had 1 atrial lead in high right atrial septal and 2 ventricular leads (SelectSecure lead and defibrillator lead) in LBBA and right ventricular apical positions.

† Dual-chamber pacemakers in the CP group had atrial and ventricular leads in the right atrial appendage and right ventricular apical positions.

§ CRT-P and CRT-D devices in the CP group had atrial and left ventricular leads in the high right atrial septal and coronary sinus positions.

### Procedural and lead parameters

There were no significant differences observed for procedural times (95.0 ± 31.4 minutes vs 86.5 ± 33.3 minutes; *P* = .185) or fluoroscopy times (12.1 ± 4.5 minutes vs 12.3 ± 13.5 minutes; *P* = .887) between the PP and CP cohorts (Table 2). However, after excluding cardiac resynchronization devices (20 patients: 4 in the PP cohort and 16 in the CP cohort), procedural and fluoroscopy times (11.5 ± 4.5 minutes and 7.5 ± 4.4 minutes, respectively) were significantly shorter in the control cohort (Table 2).

**Table 2:** Procedural outcomes and follow-up characteristics

	All	PP	CP	*P* value	PP excluding CRT	CP excluding CRT	*P* value
	(n = 96)	(n = 32)	(n = 64)		(n = 28)	(n = 48)	
Procedural parameters							
Procedural time (min)	89.4 ± 31.4	95.0 ± 31.4	86.5 ± 33.3	.185	89.8 ± 22.1	72.6 ± 18.2	.0011
Fluoroscopy time (min)	12.2 ± 11.2	12.1 ± 4.5	12.3 ± 13.5	.887	11.5 ± 4.5	7.5 ± 4.4	<.001
Air Kerma (mGy)	60.0 ± 73.3	76.5 ± 81.2	51.6 ± 67.5	.146	80.9 ± 87.5	36.6 ± 61.6	.0117
Postimplant parameters							
Atrial parameters							
Sensing (mV)	3.2 ± 2.1	1.8 ± 0.8	3.8 ± 2.3	<.001	1.8 ± 0.8	4.0 ± 2.3	<.001
Pacing threshold (V)	1.0 ± 0.7	1.5 ± 1.1	0.8 ± 0.3	<.001	1.5 ± 1.1	0.8 ± 0.3	<.001
Impedance (Ω)	643.9 ± 156.7	756.5 ± 194.9	593.0 ± 100.8	<.001	755.6 ± 204.4	595.7 ± 106.3	<.001
Ventricular parameters							
Sensing (mV)	12.6 ± 5.3	12.4 ± 5.9	12.6 ± 5.0	.841	12.5 ± 6.0	12.2 ± 4.9	.857
Pacing threshold (V)	0.5 ± 0.2	0.6 ± 0.2	0.5 ± 0.1	.003	0.7 ± 0.3	0.5 ± 0.1	<.001
Impedance (Ω)	834.1 ± 313.1	696.4 ± 181.2	896.3 ± 339.1	<.001	721.4 ± 178.8	891.6 ± 243.3	.00304
Final follow-up parameters							
Atrial parameters							
Sensing (mV)	3.8 ± 2.8	1.7 ± 0.8	4.8 ± 2.8	<.001	1.8 ± 0.8	4.8 ± 3.1	<.001
Pacing threshold (V)	0.8 ± 0.5	1.2 ± 0.8	0.7 ± 0.3	.006	1.2 ± 0.8	0.7 ± 0.3	<.001
Impedance (Ω)	632.2 ± 134.2	592.5 ± 145.8	650.4 ± 124.4	.0857	581.1 ± 138.3	663.9 ± 121.2	.0132
Percentage pacing	29.7 ± 31.6	33.8 ± 34.3	28.1 ± 30.3	.511	36.9 ± 35.4	30.7 ± 32.5	.495
Ventricular parameters							
Sensing (mV)	16.3 ± 6.5	16.3 ± 7.1	16.3 ± 6.1	.987	16.4 ± 7.4	15.7 ± 5.8	.713
Pacing threshold (V)	0.7 ± 0.2	0.7 ± 0.2	0.7 ± 0.2	.393	0.7 ± 0.2	0.6 ± 0.2	.669
Impedance (Ω)	699.0 ± 240.7	534.8 ± 74.2	774.1 ± 252.9	<.001	539.1 ± 71.7	788.7 ± 233.6	<.001
Percentage pacing	65.0 ± 43.8	82.2 ± 33.4	58.4 ± 45.5	.0153	81.2 ± 35.7	46.9 ± 46.8	.0052

Values are given as mean ± SD unless otherwise indicated.

CP = conventional pacing; CRT = cardiac resynchronization therapy; PP = physiological pacing.

#### Atrial lead details

Of the 32 patients in the study group (PP), all had the 3830 lead implanted in the HRAS. A total of 29 of the 32 patients had ECG data deemed adequate for assessment of P-wave morphology during pacing. Four patients were excluded due to very-low-amplitude P-wave amplitude (history of biatrial maze procedure, underlying atrial fibrillation, and poor-quality tracings). Of the assessable cohort, 21 of 29 patients (72.4%) met all 3 criteria for Bachmann pacing (Figure 2).

**Figure 3:**
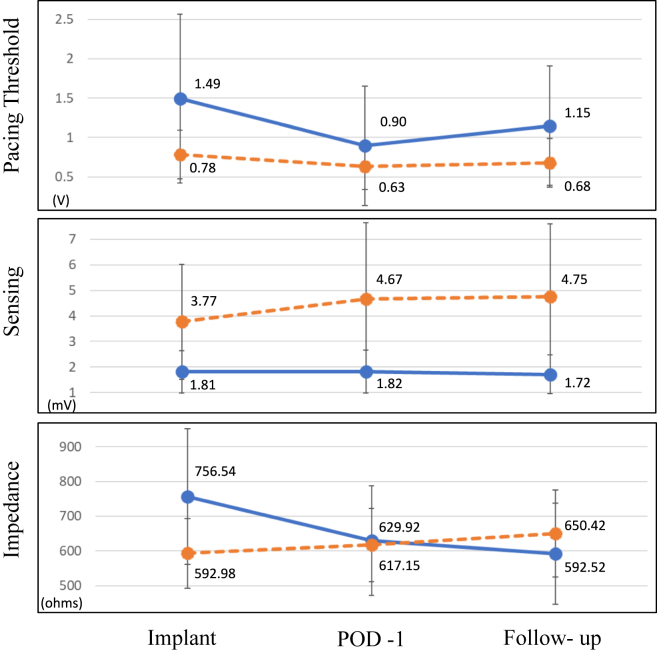
Mean pacing threshold **(top),** P-wave amplitude (sensing) **(middle),** and bipolar impedance **(bottom)** values of the physiological pacing (PP) and conventional pacing (CP) cohorts at 3 follow-up points: day of implant, first postoperative day (POD-1), and final follow-up. *Blue* denotes PP cohort; *orange* denotes CP cohort.

In the 64 patients in the CP group, 26 and 38 had active and passive fixation leads implanted, respectively. The leads implanted in the atrium in the PP cohort exhibited higher pacing thresholds (1.5 ± 1.1 mA vs 0.8 ± 0.3 mA; *P* <.001) and lower sensing (1.8 ± 0.8 mV vs 3.8 ± 2.3 mV; *P* <.001) values at the time of implantation compared to those in the CP cohort. These statistically significant differences persisted during follow-up (Table 2 and Figure 3). A comparison of the changes in lead parameters for the atrial leads between implantation and the final follow-up revealed significant differences in sensing and impedance values, but not for pacing thresholds (Table 3).

**Table 3:** Comparison of change values between postimplantation parameters and final follow-up parameters

	PP (n = 32)	CP (n = 64)	*P* value
*Atrial parameters*			
Δ Sensing (mV)	–0.04 ± 0.87	1.06 ± 2.03	<.001
Δ Pacing threshold (V)	–0.36 ± 0.87	–0.11 ± 0.39	.205
Δ Impedance (Ω)	–165.81 ± 180.63	47.28 ± 130.60	<.001
*Ventricular parameters*			
Δ Sensing (mV)	3.05 ± 4.26	3.70 ± 4.35	.563
Δ Pacing threshold (V)	0.08 ± 0.33	0.17 ± 0.20	.182
Δ Impedance (Ω)	–154.35 ± 165.75	–127.40 ± 250.88	.562

Values are given as mean ± SD unless otherwise indicated.

CP = conventional pacing; PP = physiological pacing.

Other than higher sensing (1.98 ± 0.89 mV vs 1.33 ± 0.59 mV; *P* = .0478) in cases with Bachmann capture (Bachmann pacing group [21/29]) compared to non–Bachmann capture cases (non–Bachmann pacing group [8/29]) (Supplemental Figure 1), there were no significant differences in pacing thresholds and impedances between the 2 groups (Table 4). In addition, there were no significant differences in P-wave duration in sinus rhythm as well as during atrial pacing.

**Table 4:** Comparison of follow-up between Bachmann pacing and non–Bachmann pacing in the PP group

	Bachmann pacing	Non–Bachmann pacing	*P* value
	(n = 21)	(n = 8)	
P-wave duration			
Sinus rhythm (ms)	120 ± 19	105 ± 22	.142
Pacing (ms)	96 ± 14	97 ± 16	.929
Postimplant parameters			
Atrial parameters			
Sensing (mV)	1.98 ± 0.89	1.33 ± 0.59	.0478
Pacing threshold (V)	1.60 ± 1.19	1.30 ± 0.65	.472
Impedance (Ω)	731.74 ± 189.59	793.00 ± 248.49	.569
Ventricular parameters			
Sensing (mV)	10.78 ± 5.86	15.31 ± 5.49	.0987
Pacing threshold (V)	0.63 ± 0.27	0.68 ± 0.24	.674
Impedance (Ω)	715.00 ± 126.54	719.86 ± 193.85	.952
Final follow-up parameters			
Atrial parameters			
Sensing (mV)	1.73 ± 0.75	1.73 ± 0.80	.999
Pacing threshold (V)	1.36 ± 0.83	0.75 ± 0.26	.012
Impedance (Ω)	609.1 ± 180.2	551.0 ± 29.0	.201
Percentage pacing (%)	35.1 ± 32.2	31.5 ± 43.2	.851
Ventricular parameters			
Sensing (mV)	15.16 ± 8.70	17.24 ± 2.85	.403
Pacing threshold (V)	0.65 ± 0.22	0.75 ± 0.23	.323
Impedance (Ω)	562.61 ± 67.8	475.00 ± 60.1	.00804
Percentage pacing (%)	83.89 ± 37.2	73.93 ± 33.6	.553

Values are given as mean ± SD unless otherwise indicated.

PP = physiological pacing.

#### Ventricular lead details

Of the 32 patients in the study group (PP), all had the 3830 lead implanted into the ventricular septum. Of these 32 patients, 30 (93.8%) met criteria for LBBAP and 2 of 32 patients with RV septal pacing morphology due to an inability to penetrate the septum. Mean QRS duration in patients (30/32) who achieved LBBAP was 126.6 ± 22.2 ms. Of the 64 patients in the CP group, 16 and 48 patients had active and passive fixation leads, respectively, implanted in the RV apex. Of these, 44 were pacemaker leads and 20 were defibrillator leads.

The PP cohort with LBBAP exhibited a significantly higher ventricular pacing threshold (0.6 ± 0.2 mA vs 0.5 ± 0.1 mA; *P* = .003) and lower impedance values (696.4 ± 181.2 Ω vs 896.3 ± 339.1 Ω; *P* <.001) at the time of implantation compared to those in the CP cohort, all of whom had RV apical leads (pacing as well as defibrillator leads). There were no significant differences in the sensing values between the 2 cohorts. In follow-up of the ventricular leads, the differences in impedance values persisted, but sensing values and pacing thresholds remained comparable (Table 2). A comparison of the changes in sensing and threshold values between implantation and the final follow-up also revealed no significant differences (Table 3).

### Complications

Of the entire cohort of 96 patients, lead-related complications were observed in 2 patients. One occurred in the PP cohort in whom a sudden increase in the ventricular lead pacing threshold without change in capture characteristics (nonselective LBB capture) was noted 2 weeks after implant. There was no significant change in lead impedance and sensing values, and repeat chest radiography demonstrated no change in position of the ventricular lead. Transthoracic echocardiogram did not reveal any evidence of lead perforation into the left ventricular cavity; however, after discussion with the patient, it was decided to revise the lead rather than continue observation. The second lead-related complication occurred a patient in the CP group, in whom new-onset pericardial effusion and symptoms of pericarditis were observed 6 days after implantation of a dual-chamber implantable cardioverter-defibrillator. This necessitated pericardial fluid drainage, which was noted to be sanguineous. Although no significant changes in atrial or ventricular lead parameters were noted, it was decided empirically to reposition both the atrial and ventricular leads. Both patients had an unremarkable recovery after their subsequent procedures.

## Discussion

### Legacy pacing sites

In recent years, several reports have demonstrated the advantages of LBBAP over RV apical pacing, leading to its increased adoption. However, the routine use of LBBAP for all-comers is not common practice across most operators. Potential reasons include LBBAP’s learning curve, lack of long-term efficacy and safety data, and practical workflow concerns regarding prolonging a procedure that has long been considered straightforward and routine. Although LBBAP has infrequent and unique procedural complications, the elimination of RV apical perforation and tamponade (infrequent but serious complications of RV apical leads) has not received adequate recognition. This is an important advantage given the increasing age and medical complexity of our patients. Same-day discharge for pacemaker implants, a recent trend in cardiac electrophysiological procedures, is not offered in some centers, and this may be partly driven by clinicians’ concerns regarding acute complications such as tamponade related to lead perforation, which has been reported to be as high as 0.60%.^8^

The RAA is yet another legacy site for atrial lead fixation. This site has limitations similar to those of RV apical pacing, that is, pacing site–related derangements in atrial conduction that have both acute and long-term impacts on atrial electrophysiology and function.^9^^,^^10^ Although alternate sites have been proposed over the years, the RAA has continued to remain the most common site for atrial lead fixation despite its iatrogenic conduction derangements and the potential for atrial lead perforation. The continued use of RAA pacing by clinicians likely is related to the same reasons that have been described for lack of widespread and routine adoption of LBBAP.

### Atrial septal pacing and procedural workflow

The interatrial septum has been investigated in numerous studies as an alternate site for atrial pacing. However, the report by Bailin et al^4^ distinguishes itself from other atrial septal reports in that it demonstrated a meaningful clinical outcome: reduction of AF progression. In a recent retrospective study, Infeld et al^5^ reported on HRAS pacing and described the use of specific ECG criteria to identify Bachmann bundle capture vs nonspecific septal pacing. Their report revealed that patients who achieved Bachmann pacing experienced a significant reduction in atrial arrhythmia burden, both recurrent and incident. However, this retrospective study included operators with extensive experience with this form of pacing and focused more on understanding the longer-term benefits of Bachmann pacing with respect to atrial fibrillation and less on procedural workflow. Infeld et al also reported on detailed local electrogram characteristics, both at baseline and during atrial pacing, and suggest using this to guide Bachmann pacing.

We report our initial experience using a contrast-guided, anatomic approach for selecting the pacing location along the high right atrial septum for all comers. This approach was developed based on the seminal work of Infeld et al,^5^ descriptions of Bachmann bundle anatomy, and right atrial breakout locations during biatrial flutters that use Bachmann activation.^5^^,^^11^ During this initial experience, we modified our approach by ensuring satisfactory acute sensing and pacing thresholds as opposed to primarily focusing on P-wave morphology. This learning curve may explain the higher thresholds (∼1.5 V) and lower sensing (∼1.8 mV) in our PP cohort compared to previously published data by Infeld et al, who achieved pacing thresholds of ∼ 0.78–0.92 V and 1.73–2.23 mV for sensing.^5^

We believe our high septal atrial lead implantation workflow is straightforward, and although procedural durations and fluoroscopic exposure were longer compared to the CP cohort (after excluding cardiac resynchronization therapy devices), the incremental time noted in this series remains clinically reasonable. Although we did encounter fractionated and double component atrial electrograms at pacing sites and noted isoelectric intervals preceding the P wave during threshold testing as described by Infeld et al,^5^ we believe this represents the underlying anisotropic nature of the venoatrial junction and/or lines of conduction block unique to this region. In the absence of concurrent direct recordings of the Bachmann bundle, we refrained from concluding on the relevance of these potentials in this report. We propose that by fixating the lead in the region where the Bachmann bundle inserts into the right atrium, we likely preferentially engage it as opposed to attempting to selectively or nonselectively capture it. Observations from one such case in which a multielectrode catheter was placed in the distal CS such that we could record latest atrial activation in the lateral left atrium are shown in Supplemental Figure 2. We demonstrate reduction of P-wave duration from the patient’s baseline of 142 down to 130 ms with Bachmann pacing. The coronary sinus electrograms demonstrate earlier activation of distal electrograms during Bachmann pacing compared to both sinus and RAA pacing, providing direct evidence of earlier left atrial activation.

Finally, we demonstrate that high right atrial lead fixation can be combined with LBBAP on a routine basis and that this can be performed without large increases in procedural duration and risk. In fact, the delayed development of effusion in the one patient in the CP cohort may have been avoided if routine septal pacing had been considered for both chambers.

### Study limitations

This report describes an early experience as HRAS pacing was adopted at a single institution. Its focus was on exploring the feasibility and safety of HRAS pacing as an alternative to RAA pacing for all-comers and assessing our ability to capture the Bachmann bundle. We did not pursue rigorous intraprocedural confirmation of Bachmann criteria and did not modify our approach based on local electrograms or their behavior during high- and low-output pacing. This report is significantly limited in that it has short-term follow-up; therefore, we are unable to assess the impact of this form of pacing on atrial arrhythmias or other clinical outcomes. This report also is limited by its small sample size and high usage of passive fixation leads, so claims of safety and efficacy need to be viewed with caution.

## Conclusion

High right atrial pacing can be routinely applied to patients presenting to the electrophysiology laboratory, with excellent efficacy and safety. An anatomic approach guided by fluoroscopy and contrast can result in a high proportion of Bachmann bundle capture. Furthermore, the addition of LBBAP to the above can be achieved with workflow efficiencies on par with legacy pacing workflows. This approach has the advantage of offering physiological pacing for both chambers and possible elimination of complications such as perforation-related pericarditis and tamponade. Larger confirmatory multicenter studies with longer follow-up are warranted.
