# Supplementary material for: Short-term safety and feasibility of a practical approach to combined atrial and ventricular physiological pacing: An initial single-center experience
Source: Heart Rhythm O2. 2024 May 16;5(6):357–64. doi: 10.1016/j.hroo.2024.04.002 (PMC11228114; doi:10.1016/j.hroo.2024.04.002)
Supplement: Supplemental Materials [file mmc1.docx]

**Supplemental Materials**

**Supplemental Figures and Figure Legends:**

**Supplemental Figure 1**


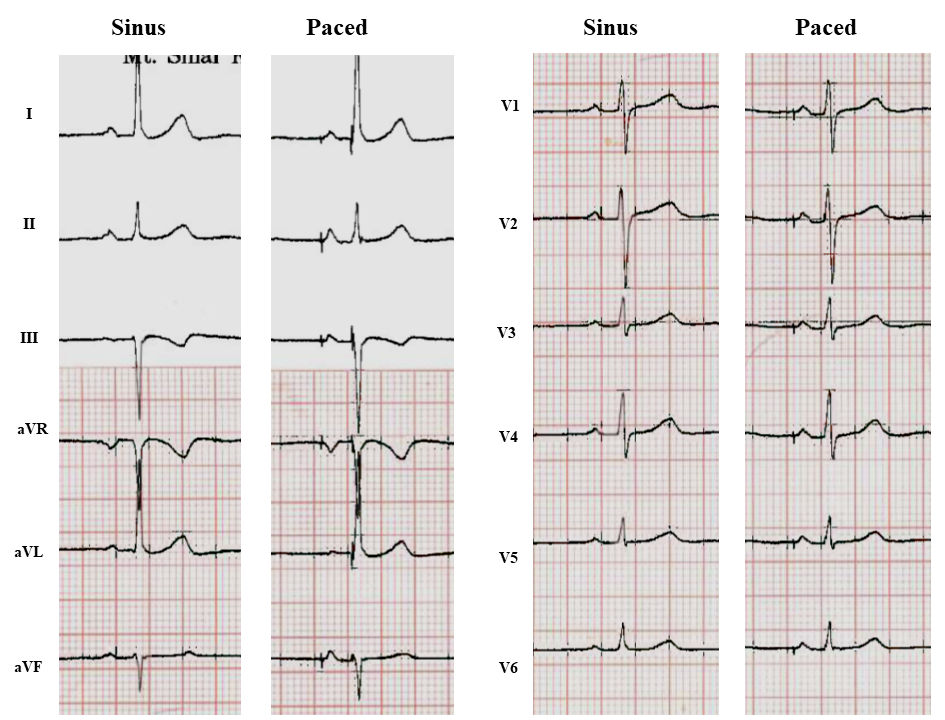


Sinus rhythm and high right atrial paced rhythm from a patient in the PP cohort who did not meet all criteria for Bachmann bundle pacing. Note the tall and narrow P waves in the inferior leads but a positive P wave in V1.

**Supplemental Figure 2**


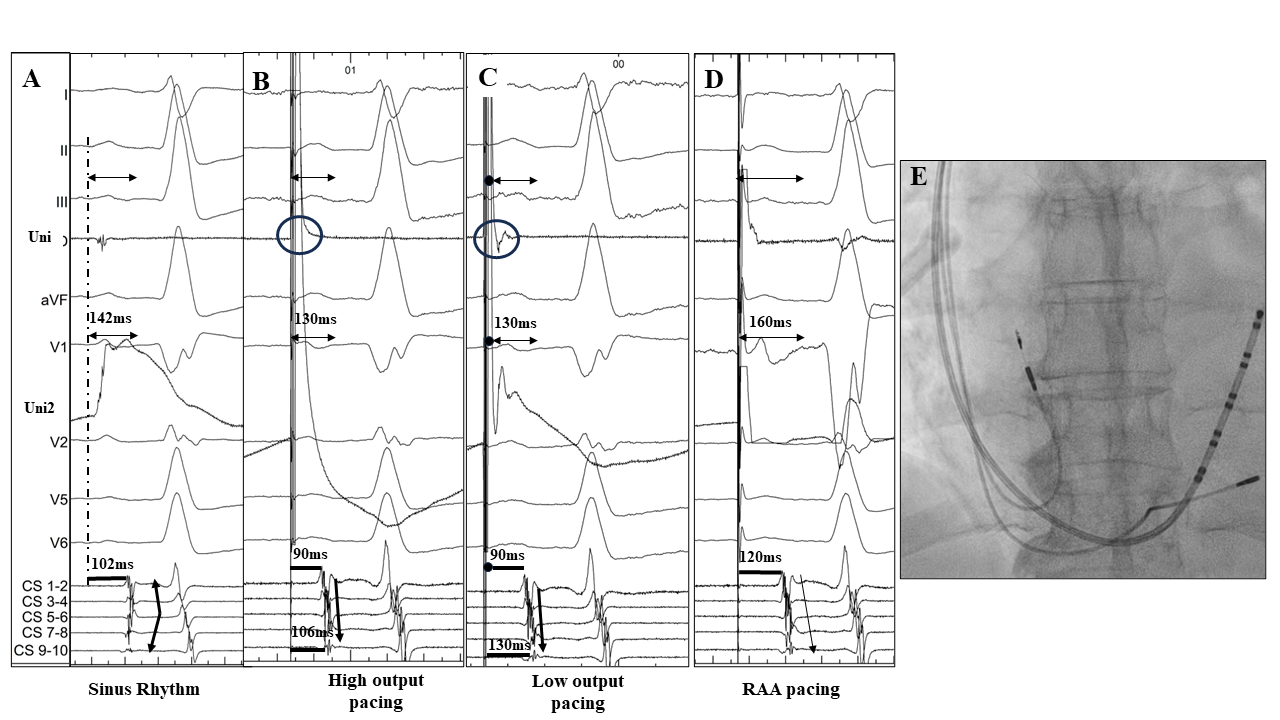
Panel A) Sinus rhythm with P wave duration of 142 msec with CS electrograms demonstrating early activation of both proximal and distal CS bipoles in reverse chevron pattern, Panel B &C) High and low output pacing from implant site with change in CS activation to linear (CS 1,2 to Cs 9,10) with shortening of ‘pacing site electrogram -CS 1,2’ duration of 90 msec. Note in Panel C- the local electrogram is not captured (Uni) allowing for a small isoelectric interval. If this interval is accounted for the paced P wave duration (130 msec) does not change, however at lower output (Panel C) there are subtle changes in P wave morphology and difference in time from Stim to CS 9, 10., Panel D) Right atrial appendage pacing with increase in P wave duration to 160 msec and lengthening of ‘pacing site electrogram -CS 1,2’ duration of 120 msec, Panel E) AP view of atrial and ventricular leads.

CS: coronary sinus

**Disclosure:**

Dr. Koruth is a consultant to Medtronic, Abbott and Boston Scientific. Unrelated to this manuscript he has equity in Affera, Farapulse, Kardium and Field Medical. He also serves as a consultant to Adagio medical and Pulse Biosciences and receives research grants from Cardiofocus, LuxMed, Medlumics and Circa Scientific.

Dr. Turagam is a consultant to Biosense Webster, and a speaker bureau to Medtronic, and Sanofi.

Dr Dukkipati has equity in Farapulse and Manual Surgical Sciences and serves as a consultant to Biosense Webster.

Dr. Reddy is a consultant to Medtronic, Abbott and Boston Scientific; and unrelated to this manuscript, he serves as a consultant for and has equity in Ablacon, Acutus Medical, Affera-Medtronic, Apama Medical-Boston Scientific, Anumana, APN Health, Aquaheart, Atacor, Autonomix, Axon Therapies, Backbeat, BioSig, CardiaCare, CardioNXT / AFTx, Circa Scientific, CoRISMA, Corvia Medical, Dinova-Hangzhou DiNovA EP Technology, East End Medical, EPD-Philips, EP Frontiers, Epix Therapeutics-Medtronic, EpiEP, Eximo, Farapulse-Boston Scientific, Field Medical, Focused Therapeutics, HRT, Intershunt, Javelin, Kardium, Keystone Heart, LuxMed, Medlumics, Middlepeak, Neutrace, Nuvera-Biosense Webster, Oracle Health, Restore Medical, Sirona Medical, SoundCath, Valcare; unrelated to this work, has served as a consultant for AtriAN, Biosense Webster, BioTel Heart, Biotronik, Cairdac, Cardiofocus, Cardionomic, CoreMap, Fire1, Gore & Associates, Impulse Dynamics, Novartis, Philips, Pulse Biosciences; and has equity in DRS Vascular, Manual Surgical Sciences, Newpace, Nyra Medical, Surecor, and Vizaramed.
